# Spray dried plasma as an alternative to antibiotics in piglet feeds, mode of action and biosafety

**DOI:** 10.1186/s40813-016-0034-1

**Published:** 2016-07-23

**Authors:** Anna Pérez-Bosque, Javier Polo, David Torrallardona

**Affiliations:** 1 Grup de Fisiologia digestiva i adaptacions nutricionals, Departament de Bioquímica i Fisiologia, Facultat de Farmàcia i Ciències de l’Alimentació, Institut de Recerca en Nutrició i Seguretat Alimentària, Universitat de Barcelona (UB), Barcelona, Spain; 2 APC Europe, S.A., Granollers, Spain; 3 IRTA, Animal Nutrition and Welfare, Mas de Bover, Ctra. Reus-El Morell, km. 3.8, E-43120 Constantí, Tarragona, Spain

**Keywords:** Antibiotics, Antibiotic replacement, Biosafety, Gut barrier function, Immunomodulation, Piglets, Spray dried plasma, Spray dried porcine plasma

## Abstract

The use of growth promoting and therapeutic antibiotics in piglet feed has been a concerning subject over the last few decades because of the risk of generating antimicrobial resistance that could be transferred to humans. As a result, many products have been proposed as potential alternatives to the use of antibiotics, and among these, spray dried plasma is considered one of the most promising. However, there have been concerns about its biosafety, particularly during periods of emergence or re-emergence of swine diseases in different regions of the world, such as the recent porcine epidemic diarrhea virus outbreak in North America. The objectives of this paper are to review recent publications about the use of spray dried plasma as an alternative to antibiotics in weaned pig diets, the possible mechanisms of action of spray dried plasma, and the existing evidence related to the biosafety of spray dried animal plasma. Particular attention is given to studies in which spray dried plasma has been directly compared to antibiotics or other alternative antimicrobial products. Several studies on the possible modes of action for spray dried plasma, such as preservation of gut barrier function or modulation of the immune response, are also reviewed. Finally, the paper focuses on the review of the existing studies on the risks of disease transmission with the use of spray dried plasma from porcine origin. Overall, spray dried plasma is a promising alternative to in-feed antimicrobials for piglets, particularly during the early stages of the post-weaning phase. Additionally, there is enough evidence to support that commercial spray dried porcine plasma is a safe product for pigs.

## Background

**Fig. 1:**
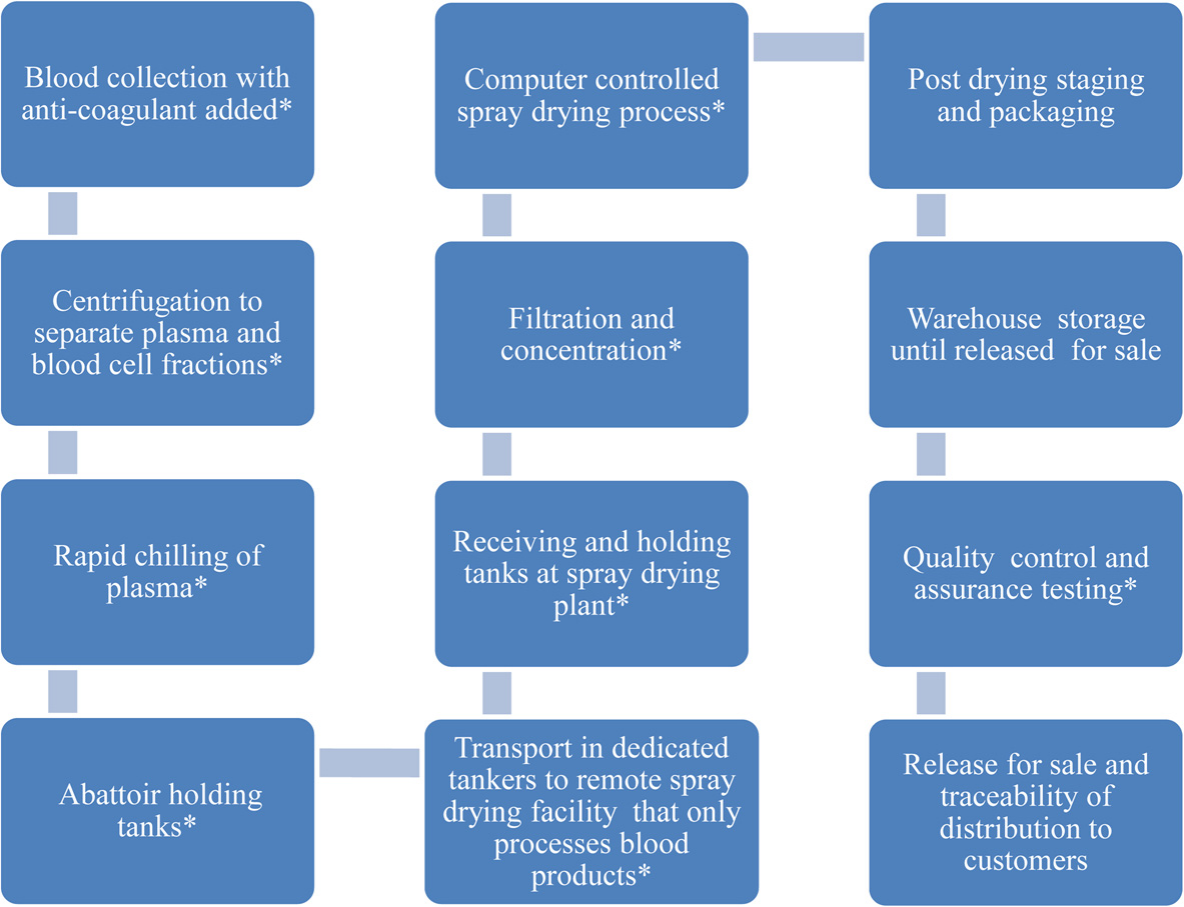
Schematic overview of general steps involved in the industrial production of spray-dried plasma. Critical control points for quality assurance and quality control. Manufacturers following good manufacturing practices collect blood from animals inspected and determined fit for slaughter for human consumption. Blood flows into an enclosed system, separated into plasma and blood cell fractions, rapidly chilled to 4 °C and held in insulated holding tanks. Then plasma is transported in cleaned, sealed dedicated tankers to a remote spray drying facility where it is held in insulated tanks. Alternatively, the whole blood is chilled to 4 °C in the slaughterhouse and transported to remote spray drying facility where it is split into plasma and blood cells fractions. At abattoirs, collection systems and holding tanks are cleaned and sanitized by standard operating procedures specific for each plant. Holding tanks at abattoirs or spray drying plants are cleaned and sanitized when emptied. Filtration and concentration systems are cleaned per standard operating procedures. Standard operating procedures are in place for continual monitoring and recording of computer controlled spray drying process. Each lot of spray dried product is identified at packaging and must pass quality control and assurance testing before release for sale. Product lot number allows traceability of distribution to customers

Spray dried plasma (SDP) is a protein rich product obtained from the industrial fractionation of blood from healthy animals. Blood is collected with an anticoagulant and centrifuged to separate the blood cells. Plasma is then concentrated and spray-dried under high pressure to achieve a minimum of 80 °C throughout its substance (Fig. 1). With this procedure, proteins preserve most of their biological activity [1, 2].

Since SDP was first proposed as a protein source for use in pig diets in the late 1980s [3, 4] many studies have demonstrated an improvement in piglet performance with its use. Inclusion levels of 4-8 % are recommended for optimal results [5–7]. Several studies have also reported that SDP reduces the incidence of post-weaning diarrhea [8–10]. A greater efficacy of SDP has been described in younger pigs which have a less mature immune system compared to older pigs fed the same diet [11], or in pigs kept under less sanitary conditions [12]. Based on these observations, the hypothesis for a protective effect of SDP by supporting the immune system or by acting directly against pathogens has gained support [12, 13].

The immunoglobulin-rich fraction in plasma has been suggested to be responsible for the beneficial effects of SDP [14]. Although newborn piglets have the capacity to absorb immunoglobulins from colostrum, this ability is lost very soon after birth, and for most of the lactation period, IgA and other immunoglobulins in milk cannot be absorbed from the gut lumen. Their presence in the lumen is thought to contribute to the defense against infectious organisms to which the sow is resistant by assisting the piglet’s innate immune response (e.g. by neutralizing toxins or by the opsonization of pathogens) [15]. However, under modern commercial conditions, piglets are usually weaned well before their ability to produce their own IgA is developed at around 6–8 weeks of age [16]. For this reason, the addition of immunoglobulins in the post-weaning diets for pigs up to 8 weeks of age may be advantageous. Many of the bioactive components in milk (including immunoglobulins, hormones and growth factors) originate from blood, cross the mammary epithelium, and are secreted intact into the milk [17, 18]. Therefore, it should be expected that many of the immunoglobulins, growth factors, bioactive peptides, and other biological components in milk may also be present in SDP.

Because of the increasing concern regarding the use of antibiotics in piglet feeds due to the risk of generating antimicrobial resistance that can be transferred to humans, intensive research has been conducted to find alternatives to both growth promoting and therapeutic antibiotics. Among the many products studied, SDP has been proposed as one of the most effective alternatives [7, 19]. However, the recent emergence of porcine epidemic diarrhea virus in North America raised concerns about the risk of disease transmission with the use of spray dried plasma from porcine origin (SDPP) in feed for pigs. The objectives of this review are to update the evidence supporting SDP as an alternative to antibiotics in diets for weaned pigs, to discuss recent studies about the possible modes of action through which it functions, and thoroughly review studies assessing the risks of disease transmission with the use of SDPP in feed.

## Review

### Efficacy of spray dried plasma and evidence supporting its use as an alternative to antibiotics

The absence of SDP in the first nursery feed has been identified as a highly significant risk factor for mortality in nursery piglets in commercial swine operations [20]. The improvements obtained in feed efficiency with the use of SDP are much more evident in piglets with a poorer health status [7], which is likely a reflection of the energy and nutrients expended to generate an immune response. Indeed, some authors have reported significant interactions between SDP and the environment on piglet performance. Thus, greater performance enhancement by SDP has been reported in piglets reared under a conventional environment compared with a clean environment (i.e. new experimental facilities) [12]. A similar interaction has also been reported for antibiotics, as they have little or no effect on performance when the animals are kept under clean conditions compared with an environment with poor sanitation [21], supporting the idea that both antibiotics and SDP may have a health promoting effect.

Most studies that evaluated dietary SDP in the presence or absence of dietary antibiotics have not found an interaction between the two products, suggesting that the effects of SDP and antibiotics may be additive [11, 12, 22, 23]. This may be explained by differences in the effectiveness of SDP and antibiotics against different pathogens. For example, in contrast to antibiotics, SDP may also be effective against viruses and toxins [24–27]. Feeding pigs with 1–2.5 % SDP during the initial weeks of the growing phase, in a farm with a high prevalence of disease associated with porcine circovirus (PCV2-SD), reduced cumulative mortality over the entire growing-finishing period from 12 to 6 % along with a five-fold reduction in medication costs per pig, even though diets contained antibiotics [24]. Additionally, it has been reported that the inclusion of SDP is effective for counteracting the negative effects of dexonivalenol (a mycotoxin produced by *Fusarium fungi*) on the feed intake and weight gain of nursery pigs [26]. In the same study, SDP was reported to be more effective than a clay binder. Recently, another study has reported that SDP, fed during the first 12 days post-weaning, mitigated the negative effects of feeding a diet containing multiple mycotoxins during the subsequent 3 weeks [27]. Finally, although some trials suggest tendencies or significant interactions between SDP and antimicrobials [28, 29], it can be hypothesized that, under the conditions of these particular studies, both types of products may have been equally effective against the specific pathogens present.

Many studies (see summary in Table 1) have shown that piglets fed SDP perform equally well or better than piglets fed antibiotics [11, 12, 23, 28–32], or other alternative products such as organic acids [30, 33], other sources of immunoglobulins [33–36], plant extracts [37], zinc oxide [33], copper sulfate [38], or carbadox [33]. In a recent study conducted under field conditions [32], the use of medicated feeds (colistin, lincomycin and spectinomycin for the first week followed by colistin and chlortetracycline during weeks 2 and 3) has been compared to the use of non-medicated feeds containing SDP (5 % for the first week and 3 % during weeks 2 and 3). Similar effects on performance were observed with both approaches, although it appeared that the effect of SDP was more effective during the first week of the trial, whereas the effect antibiotics was superior during the second and third weeks. Other studies comparing SDP with antibiotics also suggest that while the effect of SDP was larger during the first weeks post-weaning, the effect of antibiotics persists for a longer period of time [29, 39].

**Table 1:** Studies comparing spray dried plasma (SDP) to antibiotics or other alternative substances

Age of piglets (days)	Challenge	SDP dose (g/kg)	Observations for SDP vs. Control^a^	Reference Product(s) and dose (g/kg)	Observations for SDP vs. Reference Product^b^	Reference
21	Uncleaned nursery	30	No differences observed	Egg yolk antibodies (2)	No differences observed	[36]
		60	↑Weight gain		↑Weight gain	
21	None	50	↑Weight gain; ↑Gain:Feed ratio	Colistin (0.12) + Lincomycin (0.044) + Spectinomycin (0.044)	No differences observed	[32]
18	None	60	↑Weight gain; ↑Feed intake	Copper sulfate (0.2)	No differences observed	[38]
21	E. coli K99	60	↑Weight Gain	Colistin (0.3)	No differences observed	[30]
				Calcium formate (18)	↑Weight gain; ↑Feed intake	
21	E. coli K99	60	No differences observed	Colistin (0.3)	↑Villi height; ↑E. coli	[30]
				Calcium formate (18)	↑Villi height	
20	None	60	↑Gain:Feed ratio; ↓Monocytes; ↓Macrophages in Peyer’s patches; ↓Macrophages, B Lymphocytes and γδ + T cells in lymph nodes; ↓Lymphocyte and cell density in lamina propria	Carvacrol (0.015) + Cinnamaldehyde (0.009) + Capsicum oleoresin (0.006)	↓Macrophages in Peyer’s patches; ↓Macrophages and γδ + T cells in lymph nodes; ↓Lymphocyte and cell density in lamina propria	[37]
21	E. coli K99	60	↑Weight gain; ↑Gain:Feed ratio	Colistin (0.3)	No differences observed	[31]
26	None	40	↑Weight gain; ↑Feed intake; ↑Gain:Feed ratio	Avilamycine (0.04)	↑Weight gain; ↑Feed intake; ↑Gain:Feed ratio	[29]
21	E. coli K88	60	↑Feed intake; ↑Weight gain; ↓Specific K88 IgA; ↑Crypt depth; ↓IL-8; ↓TNF-α	Colistin (0.25) + Amoxycycline (0.5)	↑Feed intake; ↑Weight gain; ↑Crypt depth; ↓IL-8; ↓TNF-α; ↓IFN-γ	[23]
10	E. coli K88	50	↑Weight gain; ↓Scours; ↓Mortality; ↑Villi height	E. coli K88 specific egg yolk antibodies (5)	↓Villi height	[35]
		100	↑ Weight gain; ↓Scours; ↓Mortality; ↑Villi height; ↓Plasma urea N		↓Mortality; ↓Villi height	
10	E. coli K88	100	↓Plasma urea N; ↓Scours; ↓Mortality; ↓E. coli K88 shedding; ↑Villi height; ↑Villi:Crypt ratio	Carbadox (0.055)	No differences observed	[33]
				E. coli K88 specific egg yolk antibodies (5)	No differences observed	
				Zinc oxide (2.88)	↑Villi height; ↑Villi:Crypt ratio	
				Fumaric acid (0.02)	No differences observed	
24	E. coli K99	70	↑Weight gain; ↑Gain:Feed ratio; ↑Lactobacilli	Colistin (0.3)	↑E. coli	[28]
21	None	50	↑Weight gain; ↑Gain:Feed ratio	Colistin (0.15)	No differences observed	[11]
22 & 32	None	50	↑Gain:Feed ratio	Colistin (0.15)	No differences observed	[11]
30	None	50	↑Weight gain; ↑Feed intake	Chlortetracycline (0.11) + Sulfamethazine (0.11) + Penicillin (0.055) + Copper sulfate (0.25)	No differences observed	[12]

^a^Performance and health observations in studies comparing the effects of feeding spray dried plasma (SDP) versus a control diet without antimicrobial products

^b^Performance and health observations in studies comparing the effects of feeding spray dried plasma (SDP) versus the use of antibiotics or other alternative substances

Several authors have compared SDP to antibiotics and other alternative antimicrobial products under conditions of experimental challenge with pathogenic organisms. A comparison between SDP and colistin in piglets experimentally challenged with *E. coli* K99 [28], revealed positive effects on performance for both products, relative to a challenged control. However, while SDP resulted in an increased number of lactobacilli in the ileum and cecum, colistin reduced *E. coli* in the ileum and cecum and enterococci in the cecum. Similarly, in another study with *E. coli* K99 challenged piglets, SDP improved performance relative to a control diet, calcium formate had no effect, and colistin had an intermediate effect [30]. In piglets experimentally challenged with *E. coli* K88, SDP has been shown to have similar efficacy to an egg yolk preparation with specific antibodies against the challenging agent [35]. Both products improved performance, preserved the integrity of the gut mucosa, reduced scours, *E. coli* K88 shedding and mortality. In another trial by the same group under similar conditions [35], SDP and egg yolk antibodies were compared to zinc oxide, fumaric acid and carbadox, and all the products reduced scours and mortality while improving the integrity of the gut mucosa. Finally, a comparison between SDP and a combination of colistin and amoxycycline using piglets challenged with *E. coli* K88 [23], showed that while both products reduced the concentration of specific *E. coli* K88 IgA, only SDP reduced intestinal inflammation by down-regulating cytokine expression.

### Mode of action of spray dried animal plasma

Maintenance of gut barrier function is critical for normal nutrient absorption while excluding toxins and microorganisms [40]. Mucosal permeability depends mainly on the capacity of tight junctions to efficiently seal the apical poles of epithelial cells. The permeability of the intestinal epithelium is regulated by several stimuli and its increase is associated with secretory diarrhea [41]. Pro-inflammatory cytokines, such as IFN-γ and TNF-α, increase epithelial permeability by reducing the expression of tight junction proteins [42, 43]. Moreover, an impaired intestinal barrier function contributes to disease pathogenesis especially when luminal antigens challenge the intestine.

Several studies performed with SDP have shown positive effects on the intestinal barrier. For example, oral supplementation of piglets with SDP ameliorated rotavirus-induced diarrhea [44] and reduced the severity of disease in calves exposed to coronavirus [45]. In rats with intestinal inflammation induced by Staphylococcus enterotoxin B (SEB), SDP attenuated the toxin effects on intestinal permeability as well as on tight junction protein expression [46]. The effects of SDP supplementation in reducing a toxin-induced increase in mucosal permeability may prevent the passage of microbial and food antigens into the interstitial space, thereby blocking local inflammation [47].

These effects of SDP supplementation improving intestinal tightness were observed not only during intestinal inflammation, but also in non-inflamed pigs [48]. This effect of SDP could be mediated by increased enterocyte proliferation, as shown in vitro experiments performed by Tran et al. [49]. On the other hand, SDP also modified the intestinal morphology, since supplemented pigs showed higher villous height and reduced cellularity, which was associated to lower immune activation [37]. In this line, Maijó et al. [50] demonstrated that SDP increased IL-10 production in intestinal mucosa of non-inflamed mice, which can reduce basal immune system activation.

**Fig. 2:**
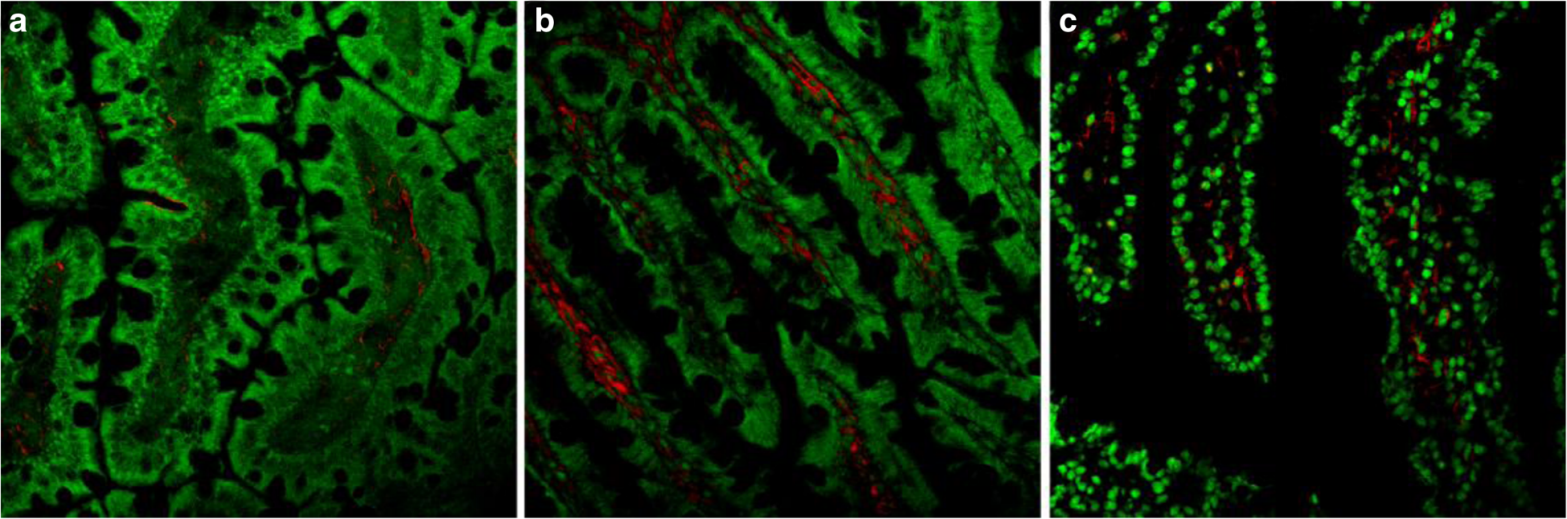
Representative images of the immunohistocheminal localization of activated lymphocytes in the jejunum of the Control (**a**), SEB (**b**) and SEB-SDP (**c**) rats. Jejunum mucosa sections were immunostained with anti-IL-2R (anti-CD25) and counterstained with Hoechst 33258 (nuclear marker). Staining for lymphocyte markers is shown in red and nuclei in green

The intestinal surface is exposed to a great variety of pathogens but also to non-pathogenic antigens. Intestinal mucosa is protected by gut-associated lymphoid tissue (GALT), which primary role is to recognize, destroy, and eliminate pathogens but must not respond to non-pathogenic antigens. However, if the immune system is stimulated and the response is not controlled, this can lead to tissue damage [51]. In this sense, the mechanism of action of SDP on pig performance involves regulation of GALT. It must be considered that SDP contains growth factors, cytokines and other biologically active compounds that may also contribute to its positive effects on pig performance [19]. These proteins can interact with immune cells present in the mucosa thus changing the cytokine environment. Bosi et al. [23] observed a reduction in the production of the pro-inflammatory cytokines (TNF-α, IL-8 and IFN-γ) in the jejunum of SDP-fed piglets challenged with enterotoxigenic *E. coli* K88. Furthermore, SDP reduced the SEB induced activation of T-helper cells in a rat model of acute intestinal inflammation [52, 53] (Fig. 2). T-helper lymphocyte activation produces a cytokine release that amplifies the immune response and inflammation [54]. These effects on the mucosal cytokine profile can explain the observed reductions in mucosal inflammation by SDP and the preventive effects of SDP on mucosal permeability and tight junction protein expression [46]. The effects of SDP on GALT are mediated by changes in the ratio between immune activation and immune tolerance [55, 56]. In particular, in SEB challenged rat undergoing mild intestinal inflammation, SDP reduced the production of mucosal pro-inflammatory cytokines and increased production of anti-inflammatory cytokine IL-10 [55], and in mice genetically modified to develop colitis spontaneously, SDP promoted the T regulatory lymphocytes [56]. This supports the hypotheses that SDP reduces over-stimulation of the mucosal immune response by enhancing IL-10 secretion and therefore, allowing more of the available energy and nutrients to be used for growth and other productive functions rather than being diverted to support the immune response.

**Fig. 3:**
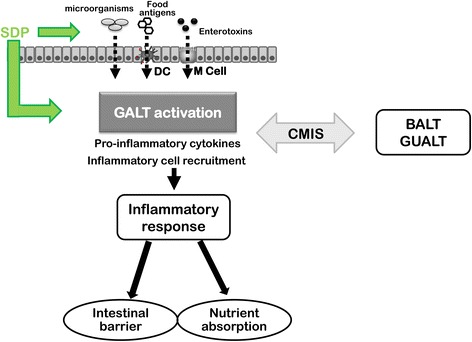
Diagram summarizing the mode of action of spray-dried plasma (SDP). Food antigens and products from commensal bacteria can enter into the intestinal lamina propria across M cells (in Peyer’s patches), by dendritic cells (DC), or by paracellular pathway. This results in activation of the Gut-Associated Lymphoid Tissue (GALT), which induces an inflammatory response that can alter different intestinal functions such as intestinal barrier or nutrient absorption. GALT is interconnected to other mucosal surfaces like the Broncoalveolar-Associated Lymphoid Tissue (BALT) or the Genito Urinary-Associated Lymphoid Tissue (GUALT) by the common mucosal immune system (CMIS). Plasma supplementation can modulate the immune response at the luminal level (e.g.: modifying the microbiota profile, reducing antigen and pathogen concentration by luminal binding). Moreover, biologically active compounds present in plasma supplements can also interact directly with mucosal immune cells. The plasma-induced changes in mucosal cytokine profile can prevent or reverse deleterious effects resulting from immune system activation

In addition to this intestinal effect, SDP also modified the immune response on other mucosal areas. For example, feeding SDP to weaned pigs challenged with swine influenza virus reduced lung lesions compared with a non-supplemented diet [25] and prevented the increased activated lymphocyte populations in a LPS induced lung inflammation [50]. Moreover, SDP supplementation also decreased the uterine concentrations of TNF-α and IFN-γ, and increased the concentration of TGF-β1 [57]. Therefore, the beneficial effects of SDP not only occur locally at the gut level, but also occur at a systemic level. Since organized GALT is an inductive site that connects with both local and peripheral effector sites (respiratory tracts, glandular tissues and the uterus mucosa), it can be further hypothesized that SDP may reduce over stimulation of the broader common mucosal immune system (Fig. 3).

### Biosafety of spray dried plasma of porcine origin

Spray dried plasma of porcine origin (SDPP) is a feed ingredient derived from the blood of pigs, and is a product whose biosafety is often under scrutiny, particularly during periods of emergence or re-emergence of swine diseases in different regions of the world. In response to these concerns, a significant amount of literature has become available to evaluate the safety of commercial SDPP about a variety of bacteria and viruses affecting the swine industry. These publications indicated several aspects in the manufacturing process of commercial SDPP that contribute to the bio-safety of this functional protein ingredient, starting with the fact that only blood from healthy pigs determined as fit for slaughter for human consumption is collected for commercially produced SDPP. This is the first critical control point in the manufacturing process of SDPP. Avoidance of collecting plasma from clinically affected pigs decreases the risk of potential pathogen transmission; however, in case of asymptomatic diseases, the safety features of the whole manufacturing process should assure inactivation of such pathogens that cannot be detected at inspection. The general procedures involve collection of pooled blood at inspected abattoirs, transport to remote processing plants, drying, and packaging of the dried product. Liquid pooled plasma used in commercial production of SDPP inherently contains antibodies with neutralizing capacity against a variety of pathogens, which contributes to the biosafety of the finished product and this is an additional critical control point in the process of manufacturing SDPP [58].

The spray-drying process has demonstrated its efficacy as a pasteurization-like process to eliminate bacteria and viruses [59]. During spray drying, it is ensured that plasma is exposed to temperatures over 80 °C throughout its substance and this is one of the most important critical control point in the manufacturing process of SDPP (Fig. 1).

Research, in which plasma has been inoculated with different viruses prior to drying, has shown that spray drying is an effective method to inactivate Pseudorabies virus, Porcine Reproductive and Respiratory Syndrome virus (PRRSV) [60], and Swine Vesicular Disease virus [61]. Animal studies have also failed to show any evidence of transmission of Porcine Circovirus type 2 (PCV2) [59, 62–64] when diets supplemented with commercial SDPP containing the genome of this virus were fed to pigs, even though these particular virus is considered to be amongst the most heat stable swine viruses known today [65]. However, other authors [66] reported PCV2 transmission in naïve pigs given an oral gavage of experimentally produced SDPP. The experimental SDPP used in this study was produced using a laboratory scale spray-dryer from the blood of a single infected pig showing PCV2 viremia without PCV2 neutralizing antibodies and clinical evidence of post-weaning multisystemic wasting syndrome. Several variables influenced these results, including high virus load (the plasma was obtained from a single pig at peak viremia), the use of low inlet (166 °C) and outlet temperatures (67-71 °C), small particle size (10 μm), and less than 1 sec residence time which is lower than those typical for industrial dryers (inlet temperature, 170-310 °C; outlet temperature, 80-90 °C; particle size, 45–150 μm; residence time, 20–90 sec). Moreover, as stated before, commercial SDPP is always obtained from healthy pigs determined as fit for slaughter for human consumption. The differences in source material and manufacturing processes may explain the contradicting results obtained between the study conducted by Patterson et al. [66] and other studies [37, 59, 62–64] that used commercially produced SDPP, even though PCV2 DNA was present in the tested commercial SDPP.

In addition, it also has been demonstrated throughout retrospective feeding studies that the manufacturing process of SDPP inactivated Hepatitis E virus [67], another virus of concern in the swine industry.

With the emergence of Porcine Epidemic Diarrhea virus (PEDV) in North America, feed containing SDPP was suspected to play a role in the spread of PEDV [68]. However, there is evidence indicating that the spray drying process, even at lower temperatures than those used to produce commercial SDPP, inactivate PEDV [69, 70]. In addition, it has also been reported that PEDV inoculated in SDPP survived for less than 7 days when stored at room temperature (20-22 °C) [69]. Similarly, other research has shown that PEDV does not survive in swine feces for more than 7 days when stored at room temperature [71]. In a recent publication [72], PEDV was inoculated in several ingredients commonly used in swine diets and stored outdoors under winter time ambient conditions of Minnesota. Samples were tested for viable PEDV using virus isolation or swine bioassay at different storage days post-inoculation (DPI). Results showed that PEDV inoculated on soybean meal remained infectious for 180 DPI, while PEDV inoculated on SDPP was not infective by swine bioassay just after 1 DPI. This study demonstrated that PEDV viability in feed appears to be influenced by ingredient type, with extended survival reported in soybean meal, and therefore pointed out a potential way of PEDV introduction in North America. Furthermore, the results confirmed that SDPP was not an ingredient where PEDV can survive for a long period of time. In addition, two studies demonstrated that feeding diets containing SDPP with the PEDV genome did not cause transmission of the disease to PEDV-naïve pigs even when fed at 5 % of the diet for either 14 [73] or 28 days [74]. Moreover, additional swine bioassays conducted with different lots of SDPP PCR positive for PEDV genome demonstrated lack of infectivity [75]. Even more, an epidemiological investigation of porcine origin feed ingredients and the occurrence of PEDV at Midwestern USA swine farms concluded that SDPP and porcine-origin feed ingredients in general, had negligible to very low association with PEDV in pigs consuming that feed [76]. Finally, it has been reported that millions of pigs in PEDV free regions remained free of PEDV even though these pig populations had been fed PEDV PCR positive SDPP [77].

The fact that only one published study [68] had associated the first cases of PEDV infection in Ontario Canada with feed containing SDPP that was PCR positive for PEDV is controversial because the origin of the infectious PEDV in that study was not clear [78]. In that paper, a bioassay reported that the SDPP used in the feed was infectious; however, the feed containing the SDPP was not infectious. Also the SDPP used in that study was manufactured more than two months earlier than the initial PEDV cases were reported [79]. As indicated in previous studies, PEDV was unable to survive spray-drying conditions typical for commercial SDPP and PEDV did not survive in SDPP samples kept in refrigerated storage for more than 2 to 3 weeks. These data suggest that the first PEDV cases reported in Canada were a consequence of cross-contamination of the SDPP product with other infective sources.

Collectively the results of these studies provide solid evidence to support the fact that commercial SDPP is a safe product for pigs, even though the genome of various swine pathogens may be present in SDPP.

Furthermore, SDPP is a dry product with low moisture (<9 %) and very low water activity (Aw <0.6). Some pathogens, especially enveloped viruses such as PRRSV, transmissible gastroenteritis virus or PEDV are not able to survive for a prolonged period in dry materials like SDPP. Therefore, as an additional safety feature, the members of the North American Spray Dried Blood & Plasma Producers Association (NASDBPP) and the European Animal Protein Association (EAPA), which represent approximately 65-70 % of the spray-dried blood products produced worldwide, voluntarily stores SDP products of porcine origin at room temperature (>20 °C) for two weeks after packaging as additional safety feature to assure customers inactivation of any potential post-processing contamination of pathogens on packaged product.

In addition, a worldwide leader spray dried plasma producer has recently introduced a new technology based on ultraviolet (UV) irradiation of liquid plasma before concentration and spray-drying that will be considered as a redundant safety step. UV irradiation of liquid plasma has demonstrated to no affect functionality of SDPP when supplemented in post-weaning diets and UV irradiation is a recognized technology able to inactivate a variety of pathogen including a diversity of bacteria and viruses, including porcine parvovirus, a model virus of very high heat and chemical resistant virus [80].

## Conclusions

There is clear evidence supporting SDP as an effective alternative to antibiotics for piglets, particularly during the first weeks post-weaning. In general, SDP had better or equivalent efficacy on pig performance compared with antibiotics or other alternative antimicrobial products. The efficacy of SDP in animal feed appears to be related mainly to an improved barrier function of the gut mucosa and the modulation of the mucosal immune response. The additive responses observed when SDP is offered in combination with antibiotics or other alternative products, suggest differences in their modes of action and this is an area that deserves further research. The available biosafety studies provide enough evidence to support that commercial SDPP is a safe product for pigs.

## Abbreviations

DPI, days post-infection; GALT, gut-associated lymphoid tissue; PCV2, Porcine Circovirus type 2; PEDV, porcine epidemic diarrhea virus; PRRSV, porcine reproductive and respiratory syndrome virus; SDP, spray dried plasma (general term for spray dried plasma independently of its source origin: porcine, bovine or mix); SDPP, spray dried porcine plasma; SEB, *S. aureus* enterotoxin B
